# Comparative idiosyncrasies in life extension by reduced mTOR signalling and its distinctiveness from dietary restriction

**DOI:** 10.1111/acel.12489

**Published:** 2016-05-03

**Authors:** Michael Garratt, Shinichi Nakagawa, Mirre J. P. Simons

**Affiliations:** ^1^ Department of PathologyUniversity of Michigan Medical SchoolAnn ArborMI48109USA; ^2^ Evolution and Ecology Research Group and School of BiologicalEarth and Environmental SciencesThe University of New South WalesSydneyNSW2052Australia; ^3^ Diabetes and Metabolism DivisionGarvan Institute of Medical ResearchSydneyNSW2010Australia; ^4^ Department of Animal and Plant SciencesUniversity of SheffieldSheffieldS10 2TNUK

**Keywords:** ageing, demography, calorie restriction, evolution, mortality, rapamycin, senescence

## Abstract

Reduced mechanistic target of rapamycin (mTOR) signalling extends lifespan in yeast, nematodes, fruit flies and mice, highlighting a physiological pathway that could modulate aging in evolutionarily divergent organisms. This signalling system is also hypothesized to play a central role in lifespan extension via dietary restriction. By collating data from 48 available published studies examining lifespan with reduced mTOR signalling, we show that reduced mTOR signalling provides similar increases in median lifespan across species, with genetic mTOR manipulations consistently providing greater life extension than pharmacological treatment with rapamycin. In contrast to the consistency in changes in median lifespan, however, the demographic causes for life extension are highly species specific. Reduced mTOR signalling extends lifespan in nematodes by strongly reducing the degree to which mortality rates increase with age (aging rate). By contrast, life extension in mice and yeast occurs largely by pushing back the onset of aging, but not altering the shape of the mortality curve once aging starts. Importantly, in mice, the altered pattern of mortality induced by reduced mTOR signalling is different to that induced by dietary restriction, which reduces the rate of aging. Effects of mTOR signalling were also sex dependent, but only within mice, and not within flies, thus again species specific. An alleviation of age‐associated mortality is not a shared feature of reduced mTOR signalling across model organisms and does not replicate the established age‐related survival benefits of dietary restriction.

## Introduction

Rapamycin and closely related analogues are considered the most promising treatments for aging and age‐related disease available for translation to humans (Johnson *et al*., 2013). Rapamycin exerts its effects by inhibiting signalling of the mechanistic target of rapamycin (mTOR) pathway. Although mTOR is present in two distinct complexes, rapamycin preferentially inhibits mTOR complex 1 (mTORC1) (Stanfel *et al*., 2009), with direct genetic reductions of mTORC1 activation also extending lifespan (e.g. Jia *et al*., 2004; Kapahi *et al*., 2004; Kaeberlein *et al*., 2005; Lamming *et al*., 2012). The physiological effects of reduced mTORC1 activation, particularly the decline in cellular growth and elevated organismal survival, are expected to be a consequence of downstream inhibitory effects on several mTORC1 substrates, particularly the ribosomal S6 kinase (S6K), which has been most intensively studied (Gems & Partridge, 2013). S6K controls protein translation and can feedback to influence other pathways linked to nutritional status, such as insulin signalling and adenosine monophosphate protein kinase (AMPK) (Selman *et al*., 2009). Decreased S6K activation is hypothesized to be a major cause of lifespan extension through genetically or pharmacologically reduced mTORC1 activation (Hansen *et al*., 2007; Gems & Partridge, 2013; Johnson *et al*., 2013).

Pharmacological treatment with rapamycin, genetic impairments of the mTORC1 complex and reduced S6K activation are all expected to extend lifespan by operating through the same pathway. Indeed, separate manipulations of each of these components have been shown to extend lifespan in the four most popular model organisms in aging research: *Saccharomyces cerevisiae* (yeast), *Caenorhabditis elegans* (nematodes), *Drosophila melanogaster* (flies) and *Mus musculus* (mice) (Johnson *et al*., 2013). Although seemingly universal across species, there are several factors that might influence the degree of lifespan extension within and between species. In both mice and flies, manipulations of mTOR signalling have been reported as sex dependent, preferentially improving female lifespan over that of males (Bjedov *et al*., 2010; Miller *et al*., 2014). In flies, one study reported that rapamycin can decrease lifespan at high concentrations (Harrison *et al*., 2010), while in mice, lifespan extension has been positively associated with rapamycin concentration (Miller *et al*., 2014). Different manipulation types (e.g. rapamycin, genetic manipulations of mTORC1 or S6K) may also have physiological effects that influence survival outside of this simplified pathway, leading to possible differences in the degree of lifespan extension: examples include the possible inhibitory effects of rapamycin on mTORC2 (Lamming *et al*., 2012), and the additional regulatory effects of mTORC1 signalling on translation initiation factors in addition to S6K (Johnson *et al*., 2013).

**Figure 1:**
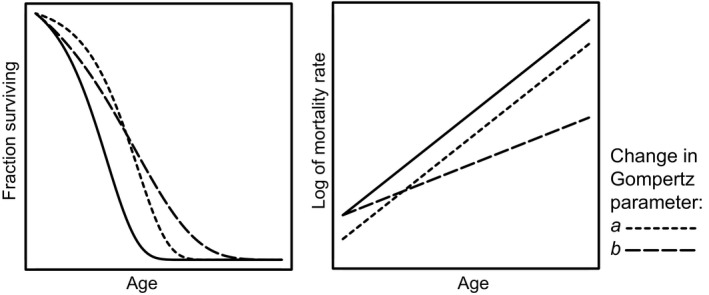
Schematic overview of the resulting survival (A) and mortality trajectories (B) when the Gompertz parameters *a* (vulnerability) and *b* (aging rate) are varied independently. Compared to a control condition (solid lines), a similar lifespan extension as measured at median lifespan through a change solely in either *a* (short dash) or *b* (long dash) is drawn.

Although manipulations of the mTOR complex can extend median lifespan, there is relatively little understanding of the demographic pathways through which this life extension occurs. Longer lifespans of animals with reduced mTOR signalling, compared to control animals, could occur because of various alterations of age‐specific mortality rate. For a manipulation to slow aging, in a demographic sense, it is expected to reduce the rate at which mortality increases in a population over time (Good & Tatar, 2001; Mair *et al*., 2003; Simons *et al*., 2013). This is usually assessed by fitting Gompertz (m(*t*) = *a* + exp(*bt*)) models (Fig. 1), which separate parameter *b*, the ‘aging rate’ of the population (the rate of increase in mortality with age), and parameter *a*, which describes the vulnerability to dying at a similar level of somatic damage (Simons *et al*., 2013; Kirkwood, 2015). Individual studies in mice have suggested that changes in either of these parameters could potentially be responsible for lifespan extension with rapamycin (Miller *et al*., 2011; Fok *et al*., 2014), although limitations in sample size in each case have hampered the ability to draw firm conclusions about the cohort under study.

To understand the consistency and demographic causes of lifespan extension via reduced mTOR signalling, we collated data from previous lifespan experiments conducted in mice, flies, yeast and nematodes, comprising data from over 30 000 individuals, and 164 different control‐treatment comparisons. We analysed this data set using meta‐analysis for both life extension – measured at median lifespan of the controls (HR_50_) – and the resulting demographic trajectory in terms of changes in mortality over different periods of life. Using such a large‐scale approach, we were able to statistically test whether lifespan extension with particular genetic manipulations mimics the mortality effects observed with pharmacological treatment with rapamycin, and even whether reduced mTOR signalling mimics the changes in mortality that occur with dietary restriction. We were also able test whether lifespan extension is similar across species – and thus whether changes in mortality in shorter‐lived and simpler organisms predict changes in longer‐lived and more complex organisms – and whether different types of manipulation provide similar survival benefits to males and females.

## Results

### Reduced mTOR signalling consistently extends lifespan

**Figure 2:**
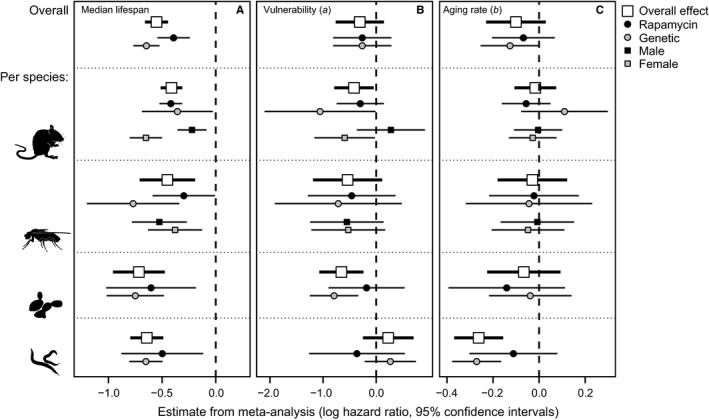
Forest plots of effect size for the logarithm of the hazard ratio (ln(HR)) for changes in median lifespan and mortality parameters with reduced mTOR signalling. Data are synthesized from 155 different control‐treatment comparisons (see supplementary data file). Bars show the effect size with 95% confidence intervals for treatment changes in median lifespan (A), vulnerability to aging (B) and rate of aging (C). Within each plot, the estimated overall effect sizes using meta‐analysis are shown across all effect sizes, separating pharmacological (rapamycin) and genetic manipulations of mTORC1 and S6K signalling, and separated per sex where applicable. Overall effects are reported across species, included species in addition to study as a random term. Overall estimates of these categories (see legend) and their 95% confidence intervals are provided, with data from all species combined, in addition to results for each individual species.

Treatment with rapamycin, genetic manipulation of components of the mTORC1 complex or deletion of the mTORC1 substrate S6K together generate an overall lifespan‐extending effect, when these collated data are analysed using meta‐analysis (HR_50_ = −0.55, −0.66: −0.44 95% CI; Fig. 2A; Tables S1 and S2). Mice, flies, nematodes and yeast show a similar level of lifespan extension in response to reduced mTOR signalling (Q_M_(df = 3) = 3.78, *P* = 0.29, Table S2). Genetic manipulation of the mTORC1 complex, or S6K (manipulation of these two targets has similar effects on lifespan, β = −0.08 ± 0.09 (SE), *P* = 0.37), generates a stronger increase in median lifespan (β = −0.25 ± 0.09 (SE), *P* = 0.006) than pharmacological treatment with rapamycin (Fig. 2A; Table S2). This greater life extension with genetic manipulations might be the result of several factors. One possibility is that rapamycin might have effects outside of reducing mTORC1 signalling that have some counteracting detrimental effect on survival, reducing the life extension of rapamycin‐treated animals to below that observed with genetic manipulations. The weaker inhibitory effects of rapamycin on mTORC2 signalling are of particular note, as reduced activity of this complex can sometimes reduce lifespan (Soukas *et al*., 2009; Lamming *et al*., 2014) and has been linked to the generation of insulin resistance in rapamycin‐treated mice (Lamming *et al*., 2012).

Smaller life extension with rapamycin compared to genetic manipulations might also be a consequence of administration of suboptimal rapamycin concentration doses (Kaeberlein, 2014), as the degree of lifespan extension can vary with concentration. The effects of different concentrations of rapamycin have only been investigated in a single study in mice (Miller *et al*., 2014), with the majority of other studies using a dietary treatment regime at a concentration of 14 ppm (Supplementary Data S1). This precludes a meta‐analysis of a concentration effect within mice. It is, however, notable that the lifespan extension achieved at the highest concentration of rapamycin employed in mice (Miller *et al*., 2014) is at least similar, if not slightly greater, than the average effect achieved through the various genetic manipulations of the TOR pathway in mice (model comparing mouse lifespan at the highest rapamycin concentration to mouse lifespan with genetic manipulation of TOR or S6K: β = −0.38 ± 0.21 (SE), *P* = 0.07). The limited data available in flies (only two studies have examined lifespan at different rapamycin concentrations) also suggest previous treatment concentrations may be suboptimal for maximizing lifespan extension, although in this case, the relationship between treatment concentration and lifespan extension is negative (β = 0.025 ± 0.011 (SE), *P* = 0.018), highlighting that rapamycin at high doses may be toxic to this species (Harrison *et al*., 2010).

### Impacts on demographic mortality are species specific

To understand how mTOR‐related pharmacological and genetic manipulations influence the demography of mortality, we calculated the Gompertz parameters for each experimental replicate using maximum likelihood (Promislow & Pletcher, 1999; Simons *et al*., 2013). Given the incongruity in concentration of rapamycin, we selected within each study the concentration that achieved the greatest life extension (based on HR_50_). Additionally, in one case (Lamming *et al*., 2012), some manipulations of different components of the mTORC1 complex did not appear to reduce mTORC1 signalling; therefore, we also excluded those specific comparisons. We took this approach because we were interested in how mTOR signalling regulates the demography of mortality, rather than in estimating an overall effect across all available effect sizes.

**Figure 3:**
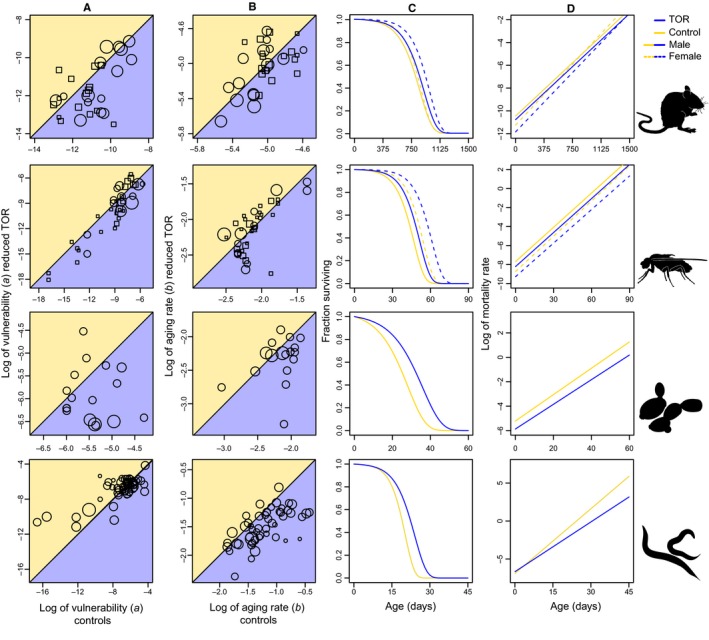
Bubble plots showing the raw data of the Gompertz parameters per species with the control plotted against reduced mTOR signalling (A and B). Bubble area reflects differences in sample size of the experimental group (circles represent males, squares represent females). Below the diagonal (shaded blue) indicates a reduction of mortality risk via this parameter. Predicted overall survival (C) and mortality trajectories (D) are provided in the right two columns. These represent the meta‐analytic mean of both Gompertz parameters of the controls (yellow lines) adjusted for the meta‐analysed hazard ratio of this parameter under reduced TOR signalling (blue lines). Dashed lines indicate females. Within mice, flies and yeast, extension in lifespan via reduced TOR signalling is a result of a demographic change in the Gompertz parameter a (i.e. frailty, vulnerability to aging, intercept), whereas in nematodes, this is the result of a change in Gompertz parameter b (i.e. aging rate, the slope of mortality).

Gompertz is a simple but effective model (Pletcher, 1999; Simons *et al*., 2013; Kirkwood, 2015) to separate the two main demographic parameters of mortality. A decline in *b*, the age‐dependent factor, is expected if a treatment reduces the rate of aging, rather than protecting against factors that reduce age‐dependent mortality across the life course (*a*: vulnerability, also known as: frailty, initial mortality rate). In a strong contrast to the effects of mTOR signalling on median lifespan, changes in both demographic parameters are largely consistent across different manipulation types but differ between species (Figs 2B,C and 3; Table S2). In nematodes, lifespan extension is associated with a strong reduction in parameter *b* (Fig. 3B), the rate of aging, without effects on parameter *a* (Fig. 3A). In mice, flies and yeast, lifespan extension is more closely associated with a reduction in parameter *a* (Fig. 3A), reducing the vulnerability to mortality across the life course. The overall predicted survival and mortality trajectories for each species are provided in Fig. 3C,D.

### Female lifespan extension is consistently greater in mice but not flies

It has been reported that reduced mTOR signalling can have a stronger lifespan impact on females than males, in both mice and flies treated with the drug rapamycin (Bjedov *et al*., 2010; Miller *et al*., 2014) and in S6K and mTORC1 component knockout mice (Selman *et al*., 2009; Lamming *et al*., 2012). It has also been noted, in mice, that the activity of the mTORC1 complex can differ between sexes (Drake *et al*., 2013; Baar *et al*., 2016) and that any concomitant reductions in mTORC2 signalling with rapamycin treatment can have greater detrimental effects on males (Lamming *et al*., 2014). We tested whether there is a consistent sex bias in lifespan extension with reduced mTOR signalling and found that the sex bias in lifespan response is species dependent (Table S3, Fig. 2A). Only in mice, but not in flies, does reduced mTOR signalling increase lifespan to a greater extent in females than males (Fig. 2A). It has been suggested that the sex‐specific response to rapamycin in mice is a consequence of differences in metabolism of this drug in males and females (Miller *et al*., 2014). However, there is no evidence for a differing effect of sex in those studies that manipulated mTOR signalling through pharmacological (rapamycin) or genetic means in mice (Table S3). This result further highlights that there are biological differences between the sexes unrelated to drug metabolism, and potentially unrelated to rapamycin's effects on mTORC2, that determine lifespan responses to reduced mTOR signalling.

### Lifespan extension by reduced mTOR signalling and DR are distinct

**Figure 4:**
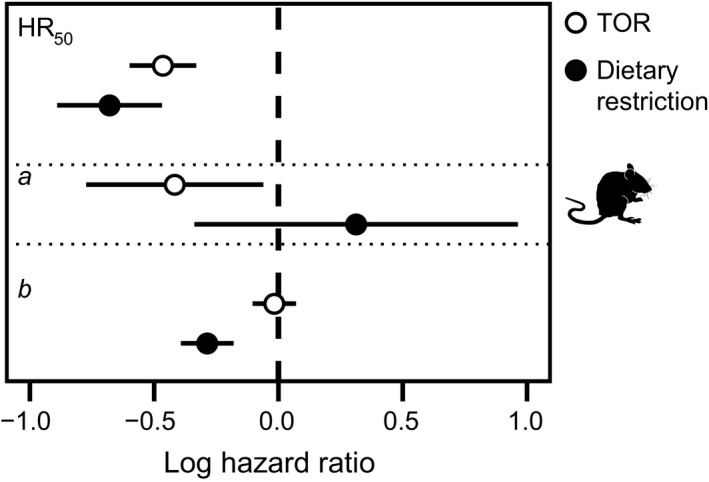
Overall effects of reduced TOR signalling (open dots) on median lifespan, vulnerability (*a*) and aging rate (*b*) in mice vs. those parameters under DR. Effects and 95% confidence intervals represent effects from separate meta‐analyses. The life extension from DR results from a change in ageing rate, vs. a change in vulnerability under reduced mTOR signalling (see text).

Reduced mTOR signalling is predicted to play a central role in lifespan extension via dietary restriction (DR) (Kennedy *et al*., 2007; Kaeberlein, 2014). However, epistasis studies in different species have produced mixed results (Kapahi *et al*., 2004; Kaeberlein *et al*., 2005; Bjedov *et al*., 2010; Ching *et al*., 2010), and in mice, rapamycin treatment and DR generate distinct changes in physiology (see discussion for examples). When comparing our results on the demographic pathways of lifespan extension with reduced mTOR signalling to a recent study examining the demographic pathway to life extension in rodents via DR (Simons *et al*., 2013), we noticed a surprisingly prominent difference: lifespan extension via reduced mTOR signalling occurs predominately through a reduction in parameter *a*, while lifespan extension via DR in rodents – although variable in its demographic response (Merry, 2005; and Fig. 4) – occurs predominately by reducing parameter *b* (Simons *et al*., 2013). Biologically, reduced mTOR signalling appears to increase resilience to aging‐related mortality, while DR reduces the increase in mortality rate that occurs as animals grow older.

To quantitatively test whether DR and reduced mTOR signalling affect mouse lifespan via differential alteration of Gompertz parameters, we combined mouse lifespan data from the current meta‐analysis with a previous meta‐analysis on DR (Simons *et al*., 2013). There was no statistical difference between the change in parameter *a* under DR and that induced with reduced mTOR signalling in mice (Q_M_(df = 1) = 1.73, *P* = 0.19; Fig. 4), despite an apparent stronger effect on *a* when mTOR is manipulated (estimates from separate models: DR: 0.31, −0.34:0.96 95% CI, mTOR: −0.42, −0.77: −0.06 95% CI). The lack of a statistical difference is probably attributable to the substantial variation in the effect of DR on this parameter (Simons *et al*., 2013); this heterogeneity is only partly explained by differences in response between strains (Table S4). By contrast, only DR consistently reduces the rate of aging (estimates from separate models: DR: −0.29, −0.39: −0.18 95% CI, mTOR: −0.016, −0.10 : 0.07 95% CI), with mice showing a significantly different response in this aging parameter depending on whether they receive a treatment that reduces mTOR signalling or whether they are dietary restricted (Q_M_(df = 1) = 8.43, *P* = 0.004). Thus, at least with regard to the way these to treatments influence age‐related mouse mortality, the effects of these treatments are distinct. This demographic perspective provides additional insight over conventional measures such as the effect on median lifespan, for which the two treatments do not significantly differ (Q_M_(df = 1) = 1.19, *P* = 0.28, Fig. 4). Notably, hardly, any heterogeneities among studies were observed in both aging parameters, *a* and *b* with reduced mTOR signalling (< 29% for both), whereas for DR, much of the variation seemed to be due to among‐study differences (*I* ^2^_[*a*]_ = 69.4% and *I* ^2^_[*b*]_ = 63.4%; Table S1). This result suggests that reduced mTOR signalling exerts much more stable and reliable life‐extending effects in mice than DR.

## Discussion

The mTORC1 signalling complex is highly responsive to nutrients, and consequently DR is expected to reduce the activity of this complex (Johnson *et al*., 2013). As reduced mTOR signalling itself extends lifespan, manipulation of this pathway has been seen as an attractive DR mimic (Longo *et al*., 2015). While both do extend lifespan, we show here that reduced mTOR signalling does not mimic the age‐related survival benefits of DR, adding to a growing picture of distinctiveness of these two lifespan‐extending interventions. This is not to say that reduced mTOR signalling is not important in the DR response – it might still be indispensable for DR‐mediated lifespan extension in mice and play an important role in the improvement of mouse survival. However, our results indicate that the differential effects of these two treatments on certain aspects of physiology (for example, insulin sensitivity (Fok *et al*., 2013), fat deposition (Fok *et al*., 2013), xenobiotic metabolism (Miller *et al*., 2014), fatty acid oxidation (Yu *et al*., 2015) and mitochondrial biology (Fok *et al*., 2014; Karunadharma *et al*., 2015)) are likely influencing at least some of the factors that influence mouse death, either their probability of occurrence or age at onset, generating these distinct mortality patterns that we observe.

Epistasis studies in mice may further help to determine whether DR provides aging benefits outside of mTOR signalling, although an epistatic interaction for lifespan does not necessarily indicate that one pathway plays a causal role in generating the survival benefits of the other, particularly if the phenotypes of the two models (e.g. DR and mTOR manipulation) are dissimilar (Hekimi *et al*., 2001). DR–mTOR epistasis studies, in other organisms, have additionally produced mixed results. In yeast and worms, DR failed to extend lifespan of either mTOR or S6K mutants (Kaeberlein *et al*., 2005), but in flies, rapamycin treatment was still able to extend the lifespan of DR individuals (Bjedov *et al*., 2010). Rapamycin treatment in flies also seems to block the negative effects that essential amino acid supplementation has on DR‐mediated life extension (Emran *et al*., 2014). While these discrepancies might partly occur because of the use of different DR regimes, which can have different gene dependencies (Greer & Brunet, 2009), it hints that the importance of mTOR in DR‐mediated lifespan extension might vary in different organisms.

The comparison of mortality effects of reduced mTOR signalling across model organisms highlights that, while generating consistent changes in average lifespan, impacts on mortality demographic parameters differ between species. In mice, yeast and (although non‐significant) flies, mTOR signalling reduced vulnerability to age‐dependent mortality, *a*. In contrast, reduced mTOR signalling in nematodes generates a strong reduction in the rate of aging, *b*. It is notable that the species examined here do not always show extended lifespan through alteration in these specific Gompertz parameters. In flies*,* genetic, dietary and temperature manipulations all seem to have treatment‐specific effects on Gompertz mortality (Pletcher, 1999), highlighting that changes in lifespan can occur through alteration in either Gompertz parameter. In nematodes, different long‐lived mutants show wide‐ranging changes in both Gompertz parameters (Johnson *et al*., 2001). Recent large‐scale demographic experiments in *C. elegans* have shown that manipulations of temperature, oxidative stress, diet, heat shock response and the insulin/IGF‐1 pathway all affect the scaling of the survival curve, analogous to vulnerability (*a*) in the Gompertz (Stroustrup *et al*., 2016). Stroustrup *et al*. suggest that this scaling pattern is therefore invariant across lifespan‐extending manipulations, but the results presented here suggest that mTOR manipulation actually has a very contrasting demographic effect in this species, warranting further direct experimental comparison. In mice, DR can influence aging rate while many genetic aging models show a change in vulnerability to age‐related death (*a*) (de Magalhães *et al*., 2005). We therefore conclude that these species‐specific responses to reduced mTOR signalling are a consequence of underlying biological differences between these model organisms in the way reduced mTOR signalling affects mortality and associated physiological pathways at different periods of life.

The specific change that is observed in the demography of mortality provides a fundamental insight into how a particular treatment influences the probability of death, both at an immediate point in time, at future time points after the treatment is discontinued, or if only started late in life. A reduction in the rate of aging, as observed with reduced mTOR signalling in *C. elegans*, is expected to be linked to permanent alterations in age‐associated damage and dysfunction (Jacobson *et al*., 2010), such that discontinuing the treatment would still leave an individual with improved survival prospects (Mair *et al*., 2003). By contrast, changes to vulnerability (*a*) are predicted to be reversible, in that current mortality is dependent on the current dietary or drug regime, without any carryover effects, as shown for DR in flies (Mair *et al*., 2003). It is notable that the degree of lifespan extension via DR in mice is strongly dependent on the age at which treatment starts, consistent with an effect on aging rate (Simons *et al*., 2013). Rapamycin treatment seems to extend lifespan to a similar degree irrespective of whether treatment starts early in life or at middle age (Miller *et al*., 2011; Table S5), consistent with the effect on the vulnerability to mortality we report here. This difference in the age dependence of these two treatments further supports the argument that these two manipulations have distinct impacts on mouse mortality and the biology of aging. Treatments that reduce age‐independent mortality in humans and mice are predicted to be able to provide the full survival benefits to an individual regardless of when the treatment starts (Vaupel *et al*., 2003; Simons *et al*., 2013). If rapamycin treatment in humans has similar stable impacts on physiology and mortality as it has shown here in mice, the health and survival benefits accrued from treatments starting late in life may still be substantial.

The meta‐analytical approach we use here, allowing comparison of lifespan extension and changes in mortality rates through genetic, dietary and pharmacological treatments, provides a comparative technique to help adjudicate the consistency (and distinctiveness) of treatments that might slow aging across species. The consistency in lifespan extension, in a demographic sense, via genetic manipulations of mTOR signalling and pharmacological treatment with rapamycin, within species, matches the view that these treatments operate through the same physiological/molecular pathway. However, the different responses across species highlight that effects on age‐specific mortality are dependent on the biological system, insights that remained hidden in single smaller sample‐sized studies on one model organism. Further focussed empirical studies will help to confirm these differential impacts on death experimentally. For example, as rapamycin treatment influenced the rate of aging in nematodes, but age‐independent mortality in yeast, experiments where rapamycin treatment is started or stopped part way through life (as conducted by Mair *et al*. (2003) and Merry *et al*. (2008) for DR in fruit flies and rats, respectively), will reveal whether these differences in Gompertz parameters of mortality do translate to permanent/transient impacts on mortality rates on an interspecies scale. Such experiments will also help to confirm whether the observed differences in mortality parameters reflect underling differences in how reduced mTOR signalling effects somatic damage and dysfunction linked to aging and vulnerability to death.

## Experimental procedures

### Search protocol

The search protocol was based on that used in previous meta‐analyses of lifespan extension (Hector *et al*., 2012; Nakagawa *et al*., 2012). Studies were including on model species considered to be wild‐type – without additional genetic manipulation or particular susceptibility to disease – and where gene expression was manipulated across all tissues. We included all genetic manipulations that inhibited the activity of the mTORC1 complex or downstream S6K. The Supplementary Data S1 shows the specific manipulation used in each study.

### Data extraction and analysis

Raw individual survival data were used whenever possible. When unavailable, mortality was measured from survival curves. Gompertz models were then fitted using maximum likelihood estimation (Pletcher, 1999), and estimates of sampling variances were obtained either from the confidence intervals around the parameters fitted on the individual level data, or estimated through simulation (Simons *et al*., 2013). To assess overall effects on lifespan, a hazard ratio at median lifespan was calculated using the number of individuals died and at risk in both experimental groups, for which sampling variances are known (Nakagawa *et al*., 2012). Meta‐analyses were run on these hazard ratios at median lifespan and the hazard ratio of the two Gompertz parameters with treatment group (reduced TOR signalling) over the control group – negative hazard ratio estimates indicate improved survival. Mixed effects meta‐analyses were conducted using the package ‘metafor’ (Viechtbauer, 2010) in R (R Core Team, 2015). Study was included as random term and because in some cases a control group was used as comparison to multiple treatment groups we modelled such data dependence using a covariance matrix. Heterogeneity in meta‐analyses was assessed by a multilevel version of *I* ^2^ (Nakagawa & Santos, 2012). We tested for publication and reporting bias (see supporting information) using rank tests of sample size against effect size and did not detect any significant bias apart from two cases (Table S6); these cases were two of 30 tests and at the expected Type I error rate (0.05). This result suggests that our meta‐analytic outcomes and conclusions are likely to be robust. Supplementary Data S2 online provide additional information.

## Conflict of interest

None declared.

## Funding

MG acknowledges support of a fellowship from the Michigan Society of Fellows. SN is supported by an ARC Future Fellowship (Australia). MJPS is supported by NERC N013832 & M005941 and Sir Henry Wellcome and Sheffield Vice Chancellor's fellowships.

## Author contributions

All authors contributed to the development of project, to the data collection and analysis, and with writing the manuscript.

## Supporting information


**Table S1**. Estimates of total heterogeneity (*I* ^2^ in %) and its sample size (the number of effect sizes, *k*) for meta‐analytic models of HR_50_, *a* (vulnerability) and *b* (aging rate) for reduced mTOR signaling (the intercept only models); for mice, the counterpart results for dietary restriction are included for *a* and *b*.
**Table S2.** Results of meta‐analytic models testing whether species (mice, flies, yeast and nematodes), manipulation type (genetic manipulation of TOR or treatment with rapamycin) or their interaction explain variance in the lifespan‐response (Hazard ratio at median lifespan, A) or change demography of mortality (Gompertz parameters, B‐C).
**Table S3.** Results of meta‐analytic models testing for sex‐dependent effects in the lifespan‐response or change in gompertz parameter in mice or flies.
**Table S4.** Proportion of variance explained by strain as an additional random term in the models.
**Table S5.** Test of the age that rapamycin treatment started within the mouse dataset.
**Table S6.** Rank tests for publication and reporting bias.

**Supplementary Data S1.** Data supplement mTOR.
**Supplementary Data S2.** Experimental procedures.

**Supplementary Data S3.** R code HR50.

**Supplementary Data S4.** R code Demography.
