# Supplementary material for: Comparative idiosyncrasies in life extension by reduced mTOR signalling and its distinctiveness from dietary restriction
Source: Aging Cell. 2016 May 3;15(4):737–43. doi: 10.1111/acel.12489 (PMC4933670; doi:10.1111/acel.12489)
Supplement: Supplementary file 1 — Table S1. Estimates of total heterogeneity (I 2 in %) and its sample size (the number of effect sizes, k) for meta‐analytic models of HR50, a (vulnerability) and b (aging rate) for reduced mTOR signaling (the intercept only models); for mice, the counterpart results for dietary restriction are included for a and b. Table S2. Results of meta‐analytic models testing whether species (mice, flies, yeast and nematodes), manipulation type (genetic manipulation of TOR or treatment with rapamycin) or their interaction explain variance in the lifespan‐response (Hazard ratio at median lifespan, A) or change demography of mortality (Gompertz parameters, B‐C). Table S3. Results of meta‐analytic models testing for sex‐dependent effects in the lifespan‐response or change in gompertz parameter in mice or flies. Table S4. Proportion of variance explained by strain as an additional random term in the models. Table S5. Test of the age that rapamycin treatment started within the mouse dataset. Table S6. Rank tests for publication and reporting bias. [file ACEL-15-737-s001.docx]

**Table S1**. Estimates of total heterogeneity (*I*^2^ in %) and its sample size (the number of effect sizes, *k*) for meta-analytic models of HR_50_, *a* (vulnerability) and *b* (aging rate) for reduced mTOR signaling (the intercept only models); for mice, the counterpart results for dietary restriction are included for *a* and *b*.

|  | *I*^2^_[HR50]_ (%) | *k*_[HR50]_ | *I*^2^_[_*_a_*_]_ (%) | *I*^2^_[_*_b_*_]_ (%) | *k*_[a and b]_ |
| --- | --- | --- | --- | --- | --- |
| Overall | 65.06 | 164 | 70.31 | 80.36 | 138 |
| Mice | < 0.01 | 49 | 7.90 | 29.56 | 33 |
| (Dietary restriction) | 66.9 | 46 | 69.47 | 63.38 | 46 |
| Fly | 85.01 | 48 | 80.06 | 87.46 | 38 |
| Yeast | 33.60 | 17 | 34.83 | 49.17 | 17 |
| Worm | 55.72 | 50 | 73.93 | 78.22 | 50 |
|  | | | | | |

**Table S2.** Results of meta-analytic models testing whether species (mice, flies, yeast and nematodes), manipulation type (genetic manipulation of TOR or treatment with rapamycin) or their interaction explain variance in the lifespan-response (Hazard ratio at median lifespan, A) or change demography of mortality (Gompertz parameters, B-C). The best fitting model (lowest AIC) and its included moderators for each test are highlighted in bold. Models were fitted using maximum likelihood to improve AIC comparison. See main text for further details.

|  |  |  |
| --- | --- | --- |
|  |  |  |
| A. Median lifespan |  |  |
| Model | Moderators | AIC |
| 1 | - | 166.0 |
| 2 | Species | 168.4 |
| **3** | **Manipulation type** | **161.0** |
| 4 | Species+Manipulation type | 165.0 |
| 5 | Species*Manipulation type | 169.3 |
| B. Change in parameter a | |  |
| Model | Moderators | AIC |
| 1 | - | 492.3 |
| **2** | **Species** | **489.6** |
| 3 | Manipulation type | 494.1 |
| 4 | Species+Manipulation type | 491.2 |
| **5** | Species*Manipulation type | 490.2 |
| C. Change in parameter b | |  |
| Model | Moderators | AIC |
| 1 | - | 87.9 |
| **2** | **Species** | **80.7** |
| 3 | Manipulation type | 86.0 |
| 4 | Species+Manipulation type | 82.3 |
| 5 | Species*Manipulation type | 81.6 |
|  |  |  |

**Table S3.** Results of meta-analytic models testing for sex-dependent effects in the lifespan-response or change in gompertz parameter in mice or flies. The best fitting model (lowest AIC) for each test is highlighted in bold. A model within mice was tested to discern whether the effect of sex was due to differential metabolism of rapamycin as this had been suggested before (see main text), but we detect no such interactive effect with manipulation type (genetic versus pharmacological manipulation of TOR).

|  |  |  |
| --- | --- | --- |
|  |  |  |
| A. Median lifespan |  |  |
| Model | Moderators | AIC |
| 1 | Manipulation type | 69.2 |
| 2 | Species + Manipulation type | 71.2 |
| 3 | Sex + Species + Manipulation type | 72.4 |
| **4** | **Sex * Species + Manipulation type** | **54.5** |
| Model Within Mice only | Moderators | AIC |
| 1 | **Sex + Manipulation type** | **31.7** |
| 2 | Sex * Manipulation type | 33.2 |
| B. Change in parameter a |  |  |
| Model | Moderators | AIC |
| 1 | Manipulation type | 410.9 |
| **2** | **Species + Manipulation type** | **412.9** |
| 3 | Sex + Species + Manipulation type | 414.6 |
| 4 | Sex * Species + Manipulation type | 411.8 |
| C. Change in parameter b |  |  |
| Model | Moderators | AIC |
| **1** | **Manipulation type** | **95.3** |
| 2 | Species + Manipulation type | 97.3 |
| 3 | Sex + Species + Manipulation type | 96.8 |
| 4 | Sex * Species + Manipulation type | 97.9 |

**Table S4.** Proportion of variance explained by strain as additional random term in the models. As tested within the null models as described in the main text and in Table S1. Conclusions from the preferred models as presented in the main text and in tables S1 and S2 were not dependent on the inclusion of strain. This is probably because strain explained very little variance in either HR_50_ and the Gompertz parameters (see below). Except fro the dietary restriction dataset in which strain explained a considerable amount of variance, but note that in this set the number of strains was substantially larger than in the rapamycin set. This could have potentially hampered estimation of study and strain specific effects, due to overlap in the levels. In other words some unique strains are from unique studies and hence study and strain cannot be separated at that level. Data are presented across all species and for mice specifically in which strain information is available and has been suggested as an important moderator before. When this estimate of variance is <0.01 this indicates no strain effect could be estimated and is likely very close to 0.

|  | **HR_50_ %** | **Gompertz a %** | **Gompertz b %** |
| --- | --- | --- | --- |
| **Across all species** | **<0.01** | **1.0** | **3.2** |
| **Mice mTOR** | **<0.01** | **<0.01** | **<0.01** |
| **Mice DR** | **49.5** | **30.1** | **35.4** |

**Table S5.** Test of the age that rapamycin treatment started within the mouse dataset. Age at start was logged, other meta-analytic procedures were employed as described above and in the main text. Effects are small and non-significant, although potentially for vulnerability a later start of treatment with rapamycin might result in a smaller effect on this parameter.

|  | **Estimate ± s.e.** | **p** |
| --- | --- | --- |
| **Median lifespan (HR_50_)** | **0.14 ± 0.11** | **0.23** |
| **Vulnerability (Gompertz *a*)** | **-0.63 ± 0.37** | **0.09** |
| **Aging rate (Gompertz *b*)** | **-0.016 ± 0.046** | **0.72** |

**Supplementary experimental procedures**

**Search protocol**

We based our search protocol on several previous studies examining the effects of dietary or drug treatments on lifespan (Hector et al., 2012; Nakagawa et al., 2012). A search was conducted on Scopus, using the search terms “(((rapamycin OR tor) AND (lifespan OR life span OR longevity OR mortality OR ageing OR aging)))”. This yielded 1132 results, which were checked for data on survival with reduced mTOR signalling. We also cross-referenced from key reviews and papers on mTOR mediated lifespan extension (Harrison et al., 2009; Johnson et al., 2013). Investigators studying mTOR and lifespan were also contacted for relevant unpublished data. The 48 studies with data that fitted the sampling criteria are listed below.

Studies were included on model species considered to be wild-type – that is, without additional genetic manipulation or particular susceptibility to disease. In studies where mTOR manipulation was combined with an additional genetic manipulation (e.g. double mutants), we included only the data from the wildtype mTOR mutant. Because we were interested in the demographic changes that occur with lifespan extension we only included studies that provided complete survival curves. As a consequence, studies that failed to show complete survival data were not included in the meta-analysis (for example, Neff et al., 2013). We were also forced to exclude studies where no minimum sample size for the lifespan cohorts was provided (for example, Villai et al., 2003), since this limited our ability to produce accurate variance estimates.

We included genetic manipulations (i.e. RNAi, deletion mutant or overexpression of dominant negative) that inhibited the activity of the mTOR complex or downstream S6K. In order to compare across studies and species we only included studies where the expression had been manipulated in a global (i.e. non-tissue specific) manner. In yeast, we included studies were mTOR activity was manipulated in the tor1 deletion mutant, and manipulation of S6K activity was achieved through knockout of the yeast ortholog Sch9. Note, that we also only included studies of replicative aging in yeast, since studies of chronological aging were usually stopped before all cells lose viability. In nematodes, we included animals heterozygous for the DAF15 mutant, an ortholog of raptor in mammals. We also included mutants of the TOR homolog Let363, and raga1 and ragc1 mutants, which facilitate mTOR activation through controlling subcellular localization (Chantranupong et al., 2015). Studies manipulating the nematode orthalog rsks-1 were included as manipulations of S6K. In flies, we included all mutants with loss of function mutations within the kinase domain of dTOR or S6K and overexpression of dominant negative versions. Mice with mutations in S6K, partial disruption of mTOR translation, and mice with a single copy of mTOR, raptor, or mlst8, or double heterozygous mutants, were included.

**Data extraction and analysis**

Raw survival data was extracted from the supplementary material or tables, or through primary authors, wherever possible. When this was not possible, mortality was measured from survival curves. In many cases these data could be extracted on the level of individual lifespan, using either symbols indicating the individual deaths or by relating the decline in survival to the reported sample sizes. In some cases, because of either large sample sizes or unknown censoring obstructing the individual level data extraction, data was extracted as population level declines in survival (Simons et al., 2013). In one case (Harrison *et al.* 2009, UM & UT Males) administration of rapamycin late in life was preceded by differential control and treatment mortality, a result from treatment allocation rather than the drug, and we solved this bias by left-censoring these deaths.

Gompertz models were fitted using maximum likelihood estimation (Pletcher, 1999), a requirement for unbiased parameter estimates (Simons et al., 2013), using ‘flexsurvreg’ from the package ‘flexsurv’ (Jackson, 2013) in R (Team, 2006). Population level declines in survival were analysed by assuming a population of 10,000 individuals to estimate the Gompertz (Simons et al., 2013). Meta-analyses require estimates of sampling variances and these were obtained from the estimated confidence intervals around the parameters fitted on the individual level data. When individual level data was not available sampling variance in the Gompertz was estimated through simulation (Simons et al., 2013). Such an approach was necessary because the sampling variances in Gompertz parameters depend on their relative magnitudes, and are not a known function of sample size as for conventional effect sizes. Using the number of individuals reported, or number of deaths reported in the case of censored data, sampling variance of the estimated Gompertz parameters was obtained per survival curve from a 1,000 simulations. To assess overall effects on lifespan a hazard ratio at median lifespan was calculated using the number of individuals died and at risk in both experimental groups, for which sampling variances are known (Nakagawa et al., 2012).

Meta-analyses were run on these hazard ratios at median lifespan and the hazard ratio of the two Gompertz parameters with treatment group (reduced TOR signalling) over the control group, and hence negative hazard ratio estimates indicate improved survival. Mixed effects meta-analyses were conducted using the package ‘metafor’ (Viechtbauer, 2010) in R (Team, 2006). Study was included as random term and because in some cases a control group was used as comparison to multiple treatment groups we modelled such data dependence using a covariance matrix. Heterogeneity in meta-analyses was assessed by a multilevel version of *I*^2^ (Nakagawa and Santos, 2012) (see supplementary material). We tested for publication and reporting bias using rank tests of sample size against effect size and did not detect considerable bias. We therefore did not employ any trim and fill methods, which would also be of limited value and reliability with the relatively small subsets of data in which some bias might be apparent.

**Publication bias**

Publication bias tests were performed using rank correlations of the average sample sizes in each study against the average effect sizes extracted (given multiple effect sizes were reported per study). We also investigated reporting bias at the effect size level correlating effect sizes extracted against their sample sizes. In such tests a significant relationship indicates a reporting bias (Nakagawa and Santos, 2012). In our case because the main effects are negative, positive correlation coefficients suggest publication or reporting bias potentially contributing to the main effect. Higher sample sizes are expected to be associated with the weaker effects should there be reporting bias of spurious results at low sample sizes. We performed these tests across all species together, and within each species.

**Table S6.** Rank tests for publication and reporting bias. In bold significant indications of reporting bias potentially contributing to an upward biased overall effect. Indications for publication bias are weak in general across the set and are unlikely to have severely biased the results reported.

|  |  | **Publication bias** | | **Reporting bias** | |
| --- | --- | --- | --- | --- | --- |
| **Parameter** | **Subset** | ***r*_s_** | **p** | ***r*_s_** | **p** |
| **Median lifespan** | Overall | 0.036 | 0.81 | 0.098 | 0.21 |
|  | Mice | 0.083 | 0.84 | 0.17 | 0.24 |
|  | Drosophila | 0.35 | 0.29 | **0.49** | **<0.01** |
|  | Yeast | -0.11 | 0.72 | -0.044 | 0.87 |
|  | C. elegans | -0.03 | 0.88 | -0.061 | 0.67 |
|  |  |  |  |  |  |
| **Gompertz *a*** | Overall | 0.02 | 0.79 | -0.062 | 0.67 |
|  | Mice | -0.045 | 0.80 | 0.067 | 0.88 |
|  | Drosophila | 0.24 | 0.145 | 0.0091 | 0.99 |
|  | Yeast | -0.45 | 0.071 | **-0.75** | **0.029** |
|  | C. elegans | -0.034 | 0.81 | -0.058 | 0.80 |
|  |  |  |  |  |  |
| **Gompertz *b*** | Overall | 0.018 | 0.83 | 0.070 | 0.64 |
|  | Mice | -0.025 | 0.89 | -0.20 | 0.61 |
|  | Drosophila | -0.14 | 0.40 | 0.34 | 0.31 |
|  | Yeast | 0.32 | 0.21 | 0.47 | 0.24 |
|  | C. elegans | 0.17 | 0.24 | 0.23 | 0.29 |

**Supplementary reference list**

Chantranupong, L., Wolfson, Rachel L., and Sabatini, David M. (2015). Nutrient-Sensing Mechanisms across Evolution. Cell *161*, 67-83.

Harrison, D.E., Strong, R., Sharp, Z.D., Nelson, J.F., Astle, C.M., Flurkey, K., Nadon, N.L., Wilkinson, J.E., Frenkel, K., Carter, C.S., et al. (2009). Rapamycin fed late in life extends lifespan in genetically heterogeneous mice. Nature *460*, 392-395.

Hector, K.L., Lagisz, M., and Nakagawa, S. (2012). The effect of resveratrol on longevity across species: a meta-analysis.

Jackson, C.H. (2013). flexible parametric survival modelling in R. Supplementary examples. (<http://cran.r-project.org/web/packages/flexsurv/index.html)>.

Johnson, S.C., Rabinovitch, P.S., and Kaeberlein, M. (2013). mTOR is a key modulator of ageing and age-related disease. Nature *493*, 338-345.

Nakagawa, S., Lagisz, M., Hector, K.L., and Spencer, H.G. (2012). Comparative and meta-analytic insights into life extension via dietary restriction. Aging Cell *11*, 401-409.

Nakagawa, S., and Santos, E.A. (2012). Methodological issues and advances in biological meta-analysis. Evol. Ecol. *26*, 1253-1274.

Neff F, Flores-Dominguez D, Ryan DP, Horsch M, Schroder S, Adler T, Afonso LC, Aguilar-Pimentel JA, Becker L, Garrett L, Hans W, Hettich MM, Holtmeier R, Holter SM, Moreth K, Prehn C, Puk O, Racz I, Rathkolb B, Rozman J, Naton B, Ordemann R, Adamski J, Beckers J, Bekeredjian R, Busch DH, Ehninger G, Graw J, Hofler H, Klingenspor M, Klopstock T, Ollert M, Stypmann J, Wolf E, Wurst W, Zimmer A, Fuchs H, Gailus-Durner V, Hrabe de Angelis M, Ehninger D (2013). Rapamycin extends murine lifespan but has limited effects on aging. J Clin Invest. 123, 3272-3291.

Pletcher (1999). Model fitting and hypothesis testing for age-specific mortality data. J. Evol. Biol. *12*, 430-439.

Simons, M.J., Koch, W., and Verhulst, S. (2013). Dietary restriction of rodents decreases aging rate without affecting initial mortality rate -- a meta-analysis. Aging Cell *12*, 410-414.

Team, R. (2006). A Language and Environment for Statistical Computing. (Austria R Foundation for Statistical Computing).

Viechtbauer, W. (2010). Conducting meta-analyses in R with the metafor package. Journal of Statistical Software *36*, 1-48.

Vellai T, Takacs-Vellai K, Zhang Y, Kovacs AL, Orosz L, Muller F (2003). Genetics: Influence of TOR kinase on lifespan in C. elegans. Nature. 426, 620-620.

**Studies included in meta-analysis**

Anisimov, V.N., Zabezhinski, M.A., Popovich, I.G., Piskunova, T.S., Semenchenko, A.V., Tyndyk, M.L., Yurova, M.N., Rosenfeld, S.V., and Blagosklonny, M.V. (2011). Rapamycin increases lifespan and inhibits spontaneous tumorigenesis in inbred female mice. Cell Cycle 10, 4230-4236.

Bjedov, I., Toivonen, J.M., Kerr, F., Slack, C., Jacobson, J., Foley, A., and Partridge, L. (2010). Mechanisms of Life Span Extension by Rapamycin in the Fruit Fly Drosophila melanogaster. Cell Metabolism 11, 35-46.

Chen, D., Li, Patrick W.-L., Goldstein, Benjamin A., Cai, W., Thomas, Emma L., Chen, F., Hubbard, Alan E., Melov, S., and Kapahi, P. (2013). Germline Signaling Mediates the Synergistically Prolonged Longevity Produced by Double Mutations in daf-2 and rsks-1 in C. elegans. Cell Reports 5, 1600-1610.

Chen, D., Thomas, E.L., and Kapahi, P. (2009a). HIF-1 Modulates Dietary Restriction-Mediated Lifespan Extension via IRE-1 in Caenorhabditis elegans. PLoS genetics 5.

Chen, X.F., Meng, F.L., and Zhou, J.Q. (2009b). Telomere recombination accelerates cellular aging in Saccharomyces cerevisiae. PLoS genetics 5.

Chin, R.M., Fu, X., Pai, M.Y., Vergnes, L., Hwang, H., Deng, G., Diep, S., Lomenick, B., Meli, V.S., Monsalve, G.C., et al. (2014). The metabolite [agr]-ketoglutarate extends lifespan by inhibiting ATP synthase and TOR. Nature 510, 397-401.

Ching, T.T., Paal, A.B., Mehta, A., Zhong, L., and Hsu, A.L. (2010). Drr-2 encodes an eIF4H that acts downstream of TOR in diet-restriction-induced longevity of C. elegans. Aging Cell 9, 545-557.

Danilov, A., Shaposhnikov, M., Plyusnina, E., Kogan, V., Fedichev, P., and Moskalev, A. (2013). Selective anticancer agents suppress aging in Drosophila. Oncotarget 4, 1507-1526.

Fok, W.C., Bokov, A., Gelfond, J., Yu, Z., Zhang, Y., Doderer, M., Chen, Y., Javors, M., Wood, W.H., Zhang, Y., et al. (2014). Combined treatment of rapamycin and dietary restriction has a larger effect on the transcriptome and metabolome of liver. Aging Cell 13, 311-319.

Ha, C.W., and Huh, W.K. (2011). Rapamycin increases rDNA stability by enhancing association of Sir2 with rDNA in Saccharomyces cerevisiae. Nucleic Acids Research 39, 1336-1350.

Hansen, M., Chandra, A., Mitic, L.L., Onken, B., Driscoll, M., and Kenyon, C. (2008). A role for autophagy in the extension of lifespan by dietary restriction in C-elegans. PLoS genetics 4.

Hansen, M., Taubert, S., Crawford, D., Libina, N., Lee, S.J., and Kenyon, C. (2007). Lifespan extension by conditions that inhibit translation in Caenorhabditis elegans. Aging Cell 6, 95-110.

Harrison, B., Tran, T.T., Taylor, D., Lee, S.D., and Min, K.J. (2010). Effect of rapamycin on lifespan in Drosophila. Geriatrics and Gerontology International 10, 110-112.

Harrison, D.E., Strong, R., Sharp, Z.D., Nelson, J.F., Astle, C.M., Flurkey, K., Nadon, N.L., Wilkinson, J.E., Frenkel, K., Carter, C.S., et al. (2009). Rapamycin fed late in life extends lifespan in genetically heterogeneous mice. Nature 460, 392-U108.

Heeren, G., Rinnerthaler, M., Laun, P., von Seyerl, P., Koessler, S., Klinger, H., Hager, M., Bogengruber, E., Jarolim, S., Simon-Nobbe, B., et al. (2009). The mitochondrial ribosomal protein of the large subunit, Afo1p, determines cellular longevity through mitochondrial back-signaling via TOR1. Aging-Us 1, 622-636.

Honda, Y., Araki, Y., Hata, T., Ichihara, K., Ito, M., Tanaka, M., and Honda, S. (2015). 10-Hydroxy-2-decenoic Acid, the Major Lipid Component of Royal Jelly, Extends the Lifespan of Caenorhabditis elegans through Dietary Restriction and Target of Rapamycin Signaling. Journal of Aging Research, 7.

Houtkooper, R.H., Mouchiroud, L., Ryu, D., Moullan, N., Katsyuba, E., Knott, G., Williams, R.W., and Auwerx, J. (2013). Mitonuclear protein imbalance as a conserved longevity mechanism. Nature 497, 451-457.

Jia, K., Chen, D., and Riddle, D.L. (2004). The TOR pathway interacts with the insulin signaling pathway to regulate C. elegans larval development, metabolism and life span. Development 131, 3897-3906.

Kaeberlein, M., Powers, R.W., Steffen, K.K., Westman, E.A., Hu, D., Dang, N., Kerr, E.O., Kirkland, K.T., Fields, S., and Kennedy, B.K. (2005). Regulation of yeast replicative life span by TOR and Sch9 in response to nutrients. Science 310, 1193-1196.

Kapahi, P., Zid, B.M., Harper, T., Koslover, D., Sapin, V., and Benzer, S. (2004). Regulation of lifespan in Drosophila by modulation of genes in the TOR signaling pathway. Current Biology 14, 885-890.

Kruegel, U., Robison, B., Dange, T., Kahlert, G., Delaney, J.R., Kotireddy, S., Tsuchiya, M., Tsuchiyama, S., Murakami, C.J., Schleit, J., et al. (2011). Elevated proteasome capacity extends replicative lifespan in saccharomyces cerevisiae. PLoS genetics 7.

Lamming, D.W., Ye, L., Katajisto, P., Goncalves, M.D., Saitoh, M., Stevens, D.M., Davis, J.G., Salmon, A.B., Richardson, A., Ahima, R.S., et al. (2012). Rapamycin-Induced Insulin Resistance Is Mediated by mTORC2 Loss and Uncoupled from Longevity. Science 335, 1638-1643.

Lapierre, L.R., Gelino, S., Melendez, A., and Hansen, M. (2011). Autophagy and Lipid Metabolism Coordinately Modulate Life Span in Germline-less C. elegans. Current Biology 21, 1507-1514.

Lin, Y.-H., Chen, Y.-C., Kao, T.-Y., Lin, Y.-C., Hsu, T.-E., Wu, Y.-C., Ja, W.W., Brummel, T.J., Kapahi, P., Yuh, C.-H., et al. (2014). Diacylglycerol lipase regulates lifespan and oxidative stress response by inversely modulating TOR signaling in Drosophila and C. elegans. Aging Cell 13, 755-764.

Lu, J.-Y., Lin, Y.-Y., Sheu, J.-C., Wu, J.-T., Lee, F.-J., Chen, Y., Lin, M.-I., Chiang, F.-T., Tai, T.-Y., Berger, Shelley L., et al. Acetylation of Yeast AMPK Controls Intrinsic Aging Independently of Caloric Restriction. Cell 146, 969-979.

Luong, N., Davies, C.R., Wessells, R.J., Graham, S.M., King, M.T., Veech, R., Bodmer, R., and Oldham, S.M. (2006). Activated FOXO-mediated insulin resistance is blocked by reduction of TOR activity. Cell Metabolism 4, 133-142.

Medvedik, O., Lamming, D.W., Kim, K.D., and Sinclair, D.A. (2007). MSN2 and MSN4 link calorie restriction and TOR to sirtuin-mediated lifespan extension in Saccharomyces cerevisiae. PLoS biology 5, e261-e261.

Meissner, B., Boll, M., Daniel, H., and Baumeister, R. (2004). Deletion of the intestinal peptide transporter affects insulin and TOR signaling in Caenorhabditis elegans. Journal of Biological Chemistry 279, 36739-36745.

Miller, R.A., Harrison, D.E., Astle, C.M., Baur, J.A., Boyd, A.R., de Cabo, R., Fernandez, E., Flurkey, K., Javors, M.A., Nelson, J.F., et al. (2011). Rapamycin, But Not Resveratrol or Simvastatin, Extends Life Span of Genetically Heterogeneous Mice. Journals of Gerontology Series a-Biological Sciences and Medical Sciences 66, 191-201.

Miller, R.A., Harrison, D.E., Astle, C.M., Fernandez, E., Flurkey, K., Han, M., Javors, M.A., Li, X., Nadon, N.L., Nelson, J.F., et al. (2014). Rapamycin-mediated lifespan increase in mice is dose and sex dependent and metabolically distinct from dietary restriction. Aging Cell 13, 468-477.

Mockett, R.J., and Nobles, A.C. (2013). Lack of robustness of life extension associated with several single-gene P element mutations in Drosophila melanogaster. J Gerontol A Biol Sci Med Sci 68, 1157-1169.

Moskalev, A.A., and Shaposhnikov, M.V. (2010). Pharmacological Inhibition of Phosphoinositide 3 and TOR Kinases Improves Survival of Drosophila melanogaster. Rejuvenation Research 13, 246-247.

Pan, K.Z., Palter, J.E., Rogers, A.N., Olsen, A., Chen, D., Lithgow, G.J., and Kapahi, P. (2007). Inhibition of mRNA translation extends lifespan in Caenorhabditis elegans. Aging Cell 6, 111-119.

Partridge, L., Alic, N., Bjedov, I., and Piper, M.D.W. (2011). Ageing in Drosophila: The role of the insulin/Igf and TOR signalling network. Experimental Gerontology 46, 376-381.

Robida-Stubbs, S., Glover-Cutter, K., Lamming, D.W., Mizunuma, M., Narasimhan, S.D., Neumann-Haefelin, E., Sabatini, D.M., and Blackwell, T.K. (2012). TOR Signaling and Rapamycin Influence Longevity by Regulating SKN-1/Nrf and DAF-16/FoxO. Cell Metabolism 15, 713-724.

Rousakis, A., Vlassis, A., Vlanti, A., Patera, S., Thireos, G., and Syntichaki, P. (2013). The general control nonderepressible-2 kinase mediates stress response and longevity induced by target of rapamycin inactivation in Caenorhabditis elegans. Aging Cell 12, 742-751.

Schieber, M., and Chandel, N.S. (2015). TOR Signaling Couples Oxygen Sensing to Lifespan in C elegans. Cell Reports *9*, 9-15.

Schreiber, M.A., Pierce-Shimomura, J.T., Chan, S., Parry, D., and McIntire, S.L. (2010). Manipulation of Behavioral Decline in Caenorhabditis elegans with the Rag GTPase raga-1. Plos Genetics 6.

Selman, C., Tullet, J.M.A., Wieser, D., Irvine, E., Lingard, S.J., Choudhury, A.I., Claret, M., Al-Qassab, H., Carmignac, D., Ramadani, F., et al. (2009). Ribosomal Protein S6 Kinase 1 Signaling Regulates Mammalian Life Span. Science 326, 140-144.

Seo, K., Choi, E., Lee, D., Jeong, D.E., Jang, S.K., and Lee, S.J. (2013). Heat shock factor 1 mediates the longevity conferred by inhibition of TOR and insulin/IGF-1 signaling pathways in C. elegans. Aging Cell 12, 1073-1081.

Sheaffer, K.L., Updike, D.L., and Mango, S.E. (2008). The Target of Rapamycin pathway antagonizes pha-4/FoxA to control development and aging. Current Biology 18, 1355-1364.

Steffen, K.K., MacKay, V.L., Kerr, E.O., Tsuchiya, M., Hu, D., Fox, L.A., Dang, N., Johnston, E.D., Oakes, J.A., Tchao, B.N., et al. (2008). Yeast life span extension by depletion of 60S ribosomal subunits is mediated by Gcn4. Cell 133, 292-302.

Sun, X.P., Komatsu, T., Lim, J., Laslo, M., Yolitz, J., Wang, C., Poirier, L., Alberico, T., and Zou, S. (2012). Nutrient-dependent requirement for SOD1 in lifespan extension by protein restriction in Drosophila melanogaster. Aging Cell 11, 783-793.

Syntichaki, P., Troulinaki, K., and Tavernarakis, N. (2007). eIF4E function in somatic cells modulates ageing in Caenorhabditis elegans. Nature *445*, 922-926.

Tóth, M.L., Sigmond, T., Borsos, É., Barna, J., Erdélyi, P., Takács-Vellai, K., Orosz, L., Kovács, A.L., Csikós, G., Sass, M., et al. (2008). Longevity pathways converge on autophagy genes to regulate life span in Caenorhabditis elegans. Autophagy 4, 330-338.

Villa-Cuesta, E., Fan, F., and Rand, D.M. (2014). Rapamycin reduces Drosophila longevity under low nutrition. Journal of Pharmacy *8*, 43-51.

Wu, J., Liu, J., Chen, E., Wang, J., Cao, L., Narayan, N., Fergusson, M., Rovira, I., Allen, M., Springer, D., et al. (2013). Increased mammalian lifespan and a segmental and tissue-specific slowing of aging after genetic reduction of mTOR expression. Cell Reports 4, 913-920.

Zhang, Y., Bokov, A., Gelfond, J., Soto, V., Ikeno, Y., Hubbard, G., Diaz, V., Sloane, L., Maslin, K., Treaster, S., et al. (2014). Rapamycin extends life and health in C57BL/6 mice. J Gerontol A Biol Sci Med Sci 69, 119-130.
